# Cladodes of *Opuntia ficus-indica* (L.) Mill. Possess Important Beneficial Properties Dependent on Their Different Stages of Maturity

**DOI:** 10.3390/plants13101365

**Published:** 2024-05-14

**Authors:** Jessica Maiuolo, Saverio Nucera, Maria Serra, Rosamaria Caminiti, Francesca Oppedisano, Roberta Macrì, Federica Scarano, Salvatore Ragusa, Carolina Muscoli, Ernesto Palma, Vincenzo Mollace

**Affiliations:** 1 IRC-FSH Center, Department of Health Sciences, University “Magna Græcia” of Catanzaro, Germaneto, 88100 Catanzaro, Italy; saverio.nucera@hotmail.it (S.N.); maria.serra@studenti.unicz.it (M.S.); rosamariacaminiti4@gmail.com (R.C.); oppedisanof@libero.it (F.O.); robertamacri85@gmail.com (R.M.); federicascar87@gmail.com (F.S.); muscoli@unicz.it (C.M.); palma@unicz.it (E.P.); mollace@libero.it (V.M.); 2 PLANTA/Research, Documentation and Training Center, Via Serraglio Vecchio 28, 90123 Palermo, Italy; sragusa@unicz.it; 3 Fondazione R. Dulbecco, 88046 Lamezia Terme, Italy

**Keywords:** *Opuntia ficus-indica*, cladodes, polyphenols, antioxidant activity, anti-apoptotic property

## Abstract

*Opuntia ficus-indica* (L.) Mill. belongs to the Cactaceae family and the genus *Opuntia;* it is a succulent plant that adapts to extreme climatic conditions. The aerial part of the plant consists of the cladodes, morphological changes of branches that appear green, are covered with thorns, and are essential to reduce excessive perspiration of water. The composition of cladodes is very varied, and the main constituents are water, fibers, polysaccharides, proteins, fatty acids, vitamins, sterols, minerals, and polyphenols. Polyphenols are responsible for many beneficial activities for human health, such as antioxidant, anti-inflammatory, anticancer, and nutritional properties. The purpose of this manuscript was to compare the properties of cladodes belonging to the same plant but with different stages of maturity. Relative extracts were tested both in vitro and on a cell line and antioxidant and anti-apoptotic properties were found. The antioxidant activity was tested by the Oxygen Radical Absorbance Capacity (ORAC) test, the 1,1-diphenyl-2-picrylhydrazil (DPPH) test, and the measurement of cellular accumulation of reactive oxygen species (ROS). Anti-apoptotic activity was evaluated by the annexin/PI assay and measurement of caspases 9 and 3 expression. The results obtained showed that the extracts considered possess antioxidant and anti-apoptotic properties. However, the different stages of maturity of cladodes are essential for the performance of both functions. In addition, important variations were made in the dissolution of the extracts that brought greater safety in their use. In conclusion, this manuscript provides further information on cladodes of *Opuntia ficus-indica*, which can be used as adjuvants in many human pathologies.

## 1. Introduction

*Opuntia ficus-indica* (L.) Mill. is a dicotyledonous plant of the category of Angiosperms, belonging to the Cactaceae family and the genus *Opuntia*. This plant grows preferably in areas with arid and semi-arid climates, like most tropical and subtropical plants [1]. It is a succulent plant, perennial, bushy, and very fast-growing, which can reach a height of 3–5 m [2]. The country of origin of *O. ficus-indica* is Mexico, where still today the plant is used for gastronomic but also curative purposes [3,4]. However, the geographical areas in which the plant is most present are Africa, the countries of the Mediterranean area, South and Central America, and Australia [5].

The stem is formed by morphological modifications of the branches, of globose, cylindrical, or flattened shape, called “cladodes”. They can have different sizes, depending on the species, the age of the plant, and the geographical area. The cladodes are covered with a layer of wax to limit perspiration; they are inserted on each other, giving life to the characteristic tree shape without a trunk and branches [6]. The structure of cladodes is a dense molecular network capable of water retention and absorption, which transforms these organs into “tanks” of water [7]. Instead of the leaves, on the cladodes very thin spines are formed, a few millimeters long, of yellow-brown color, very irritating, called glochids, which are valuable as a defense against many animals [8].

The flowers are hermaphroditic and rich in showy petals of intense yellow or yellow-orange color. Most flowers form on cladodes, each of which can produce up to 20–30 flowers [9]. The fruit is an ovoid or pyriform berry with a fleshy and edible pulp. The epicarp is green but assumes a variable color from yellow-orange to red, when ripe. The exterior of the epicarp is rich in pungent glochids that make harvesting difficult. The endocarp is formed by a very sweet and juicy pulp that wraps around numerous small woody seeds [10]. Finally, the root system of the genus *Opuntia* is mostly superficial: it appears fleshy and develops in width more than in depth. The roots can colonize the most inaccessible environments in search of water and minerals [11].

All parts of this plant are used for different purposes. The food industry was one of the first sectors in which *O. ficus-indica* was used. The fruits of *O. ficus indica* have a high antioxidant activity, due to the presence of vitamin C, flavonoids, and betalains [12]. The latter are pigments that give rise to the colors red-violet or yellow and their concentration depends on the state of ripeness of the fruits. Betalains are not only responsible for the color but also for the antioxidant activity in the fruit, which is greater than that of ascorbic acid [13]. Cactus flavonoids are also effective in decreasing oxidative stress markers and inhibiting Low-Density Lipoprotein (LDL) oxidation [14]. In addition, fruit can suppress carcinogenesis in several human cell lines such as bladder, cervix, ovary, blood (chronic myeloid leukemia), breast, and glioblastoma cells. Fruits have anti-inflammatory, neuroprotective, and hypolipidemic properties [15].

The fruit of *O. ficus-indica* contains many hard-coated seeds (10–15 g/100 g pulp): their oil content is equal to 7–15% of the weight of the whole seed and is used in cosmetics as organic oil to produce anti-aging products. Present in the seed are tocopherols, responsible for the antioxidant activity, but also fibers, minerals (calcium, potassium, and phosphorus), and proteins [16].

The flowers contain many colorless betalains and phenols responsible for their antioxidant, antibacterial, and antifungal activity [17].

In this manuscript, the cladodes will be the main objects of study: young cladodes are still often consumed in salads, and as vegetables, sauces, soups, snacks, and drinks; however, the fruit is consumed fresh [18]. *O. ficus-indica* cladodes extract is especially known for its function of reducing cholesterol, triglycerides, obesity, and diabetes [19]. *O. ficus-indica* cladodes stimulate a protective response in the gastric mucosa, preventing the development of ulcers induced by ethanol. Cellular protection occurs following the secretion of mucus from the gastric mucosa [20].

Numerous studies have reported that the juice of cladodes can counteract the effects generated by many toxic substances, including heavy metals [21]. Cladodes also possess neuroprotective effects, reducing excitotoxic damage caused by ischemia and inflammatory neuronal damage [22]. The extracts of cladodes have shown antiviral properties (herpes virus, influenza virus, and human immunodeficiency virus, HIV-1), inhibiting DNA and RNA replication [23].

Finally, not in order of importance, the antioxidant and anti-inflammatory effects of cladodes are as follows: they are a good source of natural antioxidant compounds, can ensure the scavenging of free radicals, increase the activity of antioxidant enzyme levels (superoxide dismutase, catalase, and glutathione peroxidase), reduce lipid peroxidation, and protect DNA strands from mutagenic electrophilic metabolites [24,25]. Gentile et al. investigated the effect of cladode components on a model of inflammation and concluded that these organs can reduce the expression of endothelial adhesion molecules [26]. The main constituents of cladodes are water, fibers, polysaccharides, proteins, fatty acids, vitamins, sterols, minerals, and polyphenols [27]. The latter are responsible for many beneficial activities for human health.

The excessive production of reactive oxygen or nitrogen species can determine the occurrence of different inflammatory stimuli with the synthesis and secretion of pro-inflammatory cytokines, such as the nuclear factor kappa B (NF-𝜅B/) or tumor necrosis factor-alpha (TNF-𝛼): polyphenols can modulate these inflammatory processes [28]. Finally, polyphenols have a protective effect against apoptosis [29] and play an important role in some degenerative pathologies [30].

The identification of the polyphenols contained in the cladodes was carried out through High-Performance Liquid Chromatography (HPLC) and, among the most-known compounds, naringin, ferulic acid, rutin, kaempferol-rutinoside, narcissin, tricin, eucomic acid, quercetin, p-coumaric acid 3-O-glucoside, piscidic acid, malic acid, and eicosanoic acid were present [31]. The chemical composition of the cladodes varies according to many factors including climatic conditions, the age of the plant, the cultivar, environmental factors, soil, and salinity, among others [32], demonstrating a purely dynamic attitude. For example, it has been shown that the concentration of calcium ions in cladodes increases with plant maturity [33], while soluble fibers, proteins, and bioactive compounds are more contained in middle-aged cladodes [34]. It has recently been confirmed that young cladodes have a high concentration of carbohydrates [35] and all these considerations show that their composition is dependent on their maturity stage.

In previous work, carried out by our research group, it was pointed out how the amount of insoluble fiber contained in the cladodes of *O. ficus-indica* varies with the harvest season of these organs [36]. With these premises, the present manuscript aims to assess whether the effects of cladodes (collected from the same plant and in the same season) with different stages of maturity, can affect some activities such as antioxidant, anti-inflammatory, and anti-apoptotic activities. To this end, smaller and younger cladodes (early cladodes, e-C), medium-sized cladodes (m-C), and older and larger cladodes (late cladodes, l-C) were compared. Finally, these extracts were also tested on a cell line (Huvec).

## 2. Results

### 2.1. Extracts Obtained from O. ficus-indica

Three extracts were obtained and tested in this manuscript:

(1) e-C obtained from smaller and younger cladodes.
(2) m-C obtained from medium-sized and age cladodes.
(3) l-C obtained from the largest and least young cladodes.

**Figure 1:**
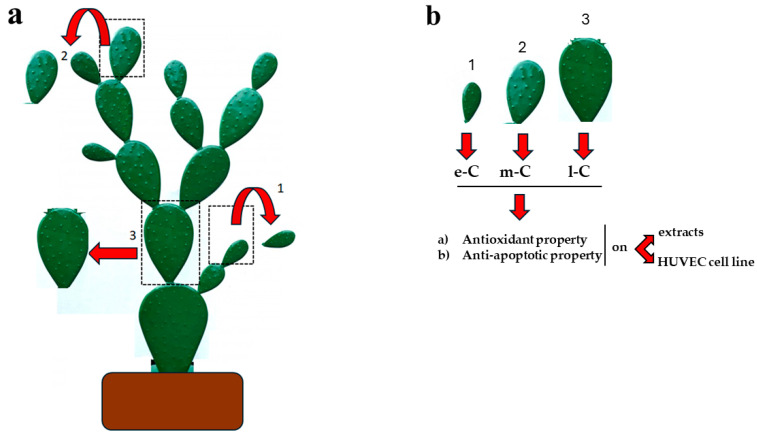
Conceptual map of the manuscript. Panel (**a**) describes the collection of cladodes from the same plant of *O. ficus-indica:* (1) a small and young cladode was chosen and collected (e-C); (2) a medium-sized and age cladode was chosen and collected (m-C); (3) a large and least young cladode was chosen and collected (l-C). Panel (**b**) highlights the continuation of work: e-C, m-C, and l-C were obtained as described and tested for antioxidant and anti-apoptotic properties.

Table 1 shows the characteristics of cladodes relative to age (expressed in days), weight (expressed in g), and length (expressed in cm), while Figure 1 highlights the conceptual map and experimental drawing of this manuscript.

### 2.2. Use of Ethanol Instead of Methanol

**Figure 2:**
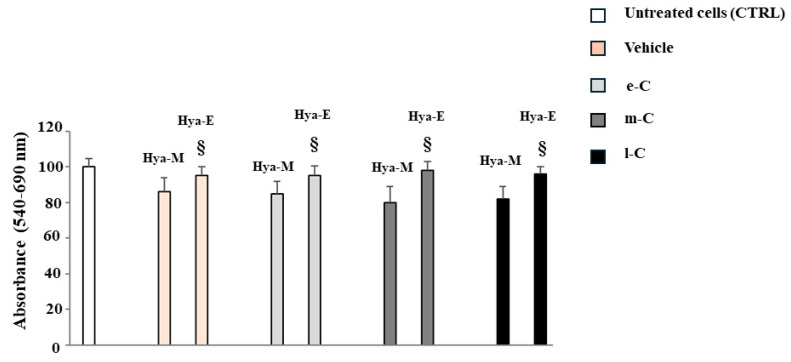
Comparison of extracts dissolved in methanol or ethanol. The extracts obtained from the cladodes of *O. ficus-indica* were dissolved in two hydroalcoholic solutions (1) water–methanol 20:80 or (2) water–ethanol 20:80, and their viability was measured through a 3-(4,5-dimethyl-2-yl)-2,5-diphenyl tetrazole bromide (MTT) test. The concentration of extracts used was 0.02 mg/mL. Untreated cells were grown in the appropriate medium and never came into contact with any alcohol. The vehicle is characterized by cells not treated with any extract, but only with the amount of hydroalcoholic solution (methanol or ethanol) used to dissolve the extracts. The hydroalcoholic solutions water–methanol and water–ethanol are indicated with Hya-M and Hya-E, respectively. e-C (early cladode), m-C (medium cladode), and l-C (late cladode) indicate the cladodes considered with different stages of maturity. Three independent experiments were performed, and the values are expressed as the mean ± sd. § denotes *p* < 0.05 vs. the counterparty dissolved in methanol. Variance analysis (ANOVA) was followed by a Tukey–Kramer comparison test.

The corresponding literature indicates that cladodes powder is dissolved in a hydroalcoholic solution of water and methanol (20:80) [37,38]. Since methanol is particularly toxic, we replaced this alcohol with ethanol. Before proceeding with this substitution, we studied the toxicity of the three extracts dissolved in methanol or ethanol, as reported in Figure 2. The observed toxicity was not attributable to the extract, as it was also present in the vehicle. Samples dissolved in ethanol showed a significant reduction in toxicity compared to those dissolved in methanol. When we dissolved the extracts in a hydroalcoholic solution (water–ethanol 20:80) the solubility was lower than that with methanol and, to make it optimal, it was necessary to raise the temperature to 30 °C.

### 2.3. Measuring the Antioxidant Potential of e-C, m-C, and l-C

#### 2.3.1. Antioxidant In Vitro Activity

**Figure 3:**
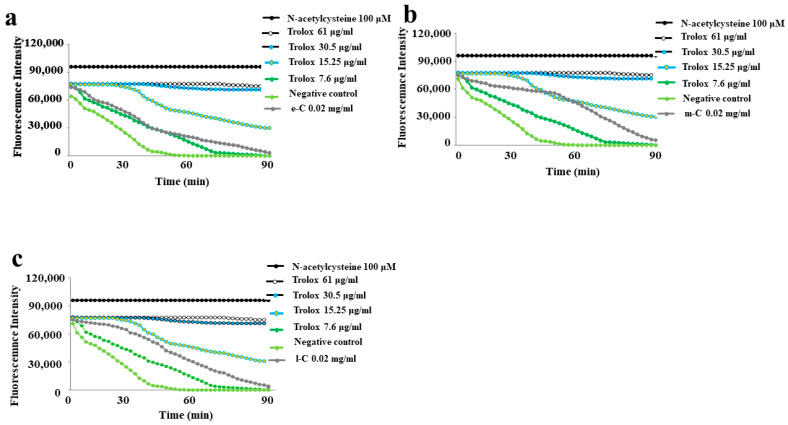
ORAC assay. The fluorescence decay curves for e-C, m-C, and l-C (from a representative experiment) are shown in the (**a**–**c**) panels, respectively.

The ORAC test found a different antioxidant rate in the e-C, m-C, and l-C and the results obtained are shown in Figure 3. In panel a, it is highlighted that the e-C extract had the lowest antioxidant activity of the three considered, and the corresponding curve is placed near the curve with a Trolox concentration of 7.6 µg/mL. The m-C extract (panel b) had a much higher antioxidant activity, and the corresponding curve is adjacent to the curve with a Trolox concentration of 15.25 µg/mL. In panel c, the l-C curve is slightly lower than the m-C.

#### 2.3.2. Radical Scavenging Effect of e-C, m-C, and l-C

**Figure 4:**
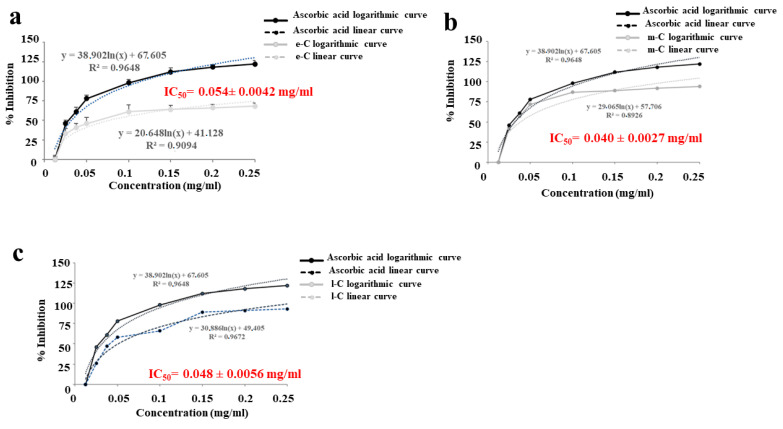
DPPH assay. Antiradical activity (% inhibition) and IC_50_ of e-C (panel **a**), m-C (panel **b**), and l-C (panel **c**) are highlighted and compared to positive control (ascorbic acid). Nonlinear regression was used to calculate IC_50_ values. A representative experiment is shown.

The results of the DPPH test (Figure 4a–c) also showed an excellent radical scavenging effect of the extracts. Different concentrations (0–0.25 mg/mL) of extracts were tested and even the lowest were in line with ascorbic acid, used as a positive control. The results are expressed as inhibition % and IC_50_ value, representing the extract concentration necessary to scavenge the 50% DPPH radicals. In addition, the antioxidant properties of the extracts were confirmed by the IC_50_ values of 0.054 ± 0.0042 mg/mL, 0.040 ± 0.0027 mg/mL, and 0.048 ± 0.0056 mg/mL for e-C, m-C, and l-C, respectively. These values showed that m-C was the best extract with the lowest IC_50_ compared to the others.

### 2.4. Effects of e-C, m-C, and l-C on Cell Viability

**Figure 5:**
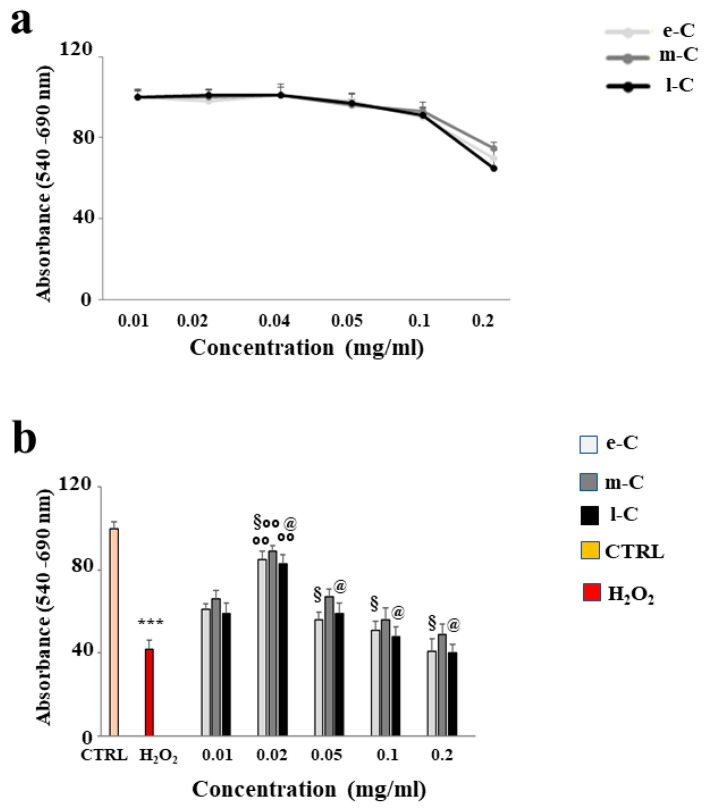
Effects of e-C, m-C, and l-C on cell viability. (**a**) Shows the curves of viability generated on Huvec cells by increasing concentrations of extracts. (**b**) Highlights the protection exerted by extracts on the damage induced by cell exposure to hydrogen peroxide (50 µM, 9 h). Three independent experiments were performed, and the values are expressed as the mean ± sd. *** denotes *p* < 0.001 vs. untreated cells; °° denotes *p* < 0.01 vs. H_2_O_2_; § denotes *p* < 0.05 vs. respective m-C. Variance analysis (ANOVA) was followed by a Tukey–Kramer comparison test.

After conducting several studies on the different extracts obtained from *O. ficus-indica,* we decided to evaluate their effects on the Huvec cell line. First, we selected a concentration that could be used without reducing cell viability. As can be seen in Figure 5a, the concentrations between 0.01 and 0.1 mg/mL of e-C, m-C, and l-C did not cause cell mortality and extracts behaved in the same way. On the contrary, only the concentration of 0.2 mg/mL showed a slight reduction in viability. As shown in panel b, the treatment with H_2_O_2_ (50 µM, 9 h) resulted in a significant reduction in cell viability, compared to untreated cells. Pre-treatment with the considered extracts, followed by exposure to H_2_O_2_, showed important variations: the concentration 0.02 mg/mL was the one that protected better than the others. In addition, once again the extract m-C had a greater action than e-C and l-C. The results obtained suggested the use of the extracts at the concentration of 0.02 mg/mL.

### 2.5. O. ficus-indica Extracts Reduce the Accumulation of ROS in the Huvec Cell Line

**Figure 6:**
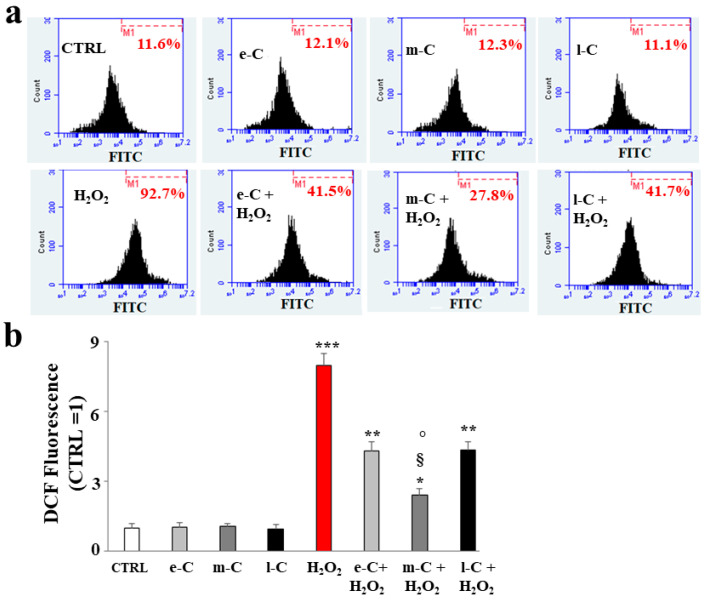
Measurement of the accumulation of ROS. In (**a**), the *x*-axis indicates the fluorescence of the fluorochrome Fluorescein-5-Isothiocyanate (FITC, connected to the probe that binds the ROS). The shift to the right indicates increased fluorescence and ROS accumulation. Hydrogen peroxide was used as a positive control (150 µM, 20 min). The *y*-axis is relative to the number of cells that we decided to acquire (20,000). (**b**) Highlights the quantification of the experiment reported in (**a**). Three independent experiments were performed, and the values are expressed as the mean ± sd. *** denotes *p* < 0.001 vs. the control; ** denotes *p* < 0.01 vs. the control; * denotes *p* < 0.05 vs. the control; § denotes *p* < 0.05 vs. e-C + H_2_O_2_. ° denotes *p* < 0.05 vs. l-C + H_2_O_2_. The Analysis of Variance (ANOVA) was followed by the Tukey–Kramer comparison test.

To evaluate the antioxidant potential of the extracts of *O. ficus-indica*, we also measured the accumulation of ROS on cell cultures pre-treated with e-C, m-C, and l-C and successively exposed to hydrogen peroxide (150 µM, 20 min). This study was carried out using cytofluorimetric readings, which allowed us to appreciate the displacement of fluorescence about the content of ROS. First, we evaluated whether the extracts alone could determine the formation of ROS and, as you can see in Figure 6a, their values are similar to that of untreated cells. On the contrary, the pre-treatment with e-C and l-C has reduced ROS generated from the exposure to H_2_O_2_, which are, however, significantly more abundant than in the untreated cells. m-C has strongly reduced ROS compared to e-C and l-C. In panel b, the respective quantification is shown.

### 2.6. Role of e-C, m-C, and l-C on Apoptosis Induced by an Inflammatory Stimulus

**Figure 7:**
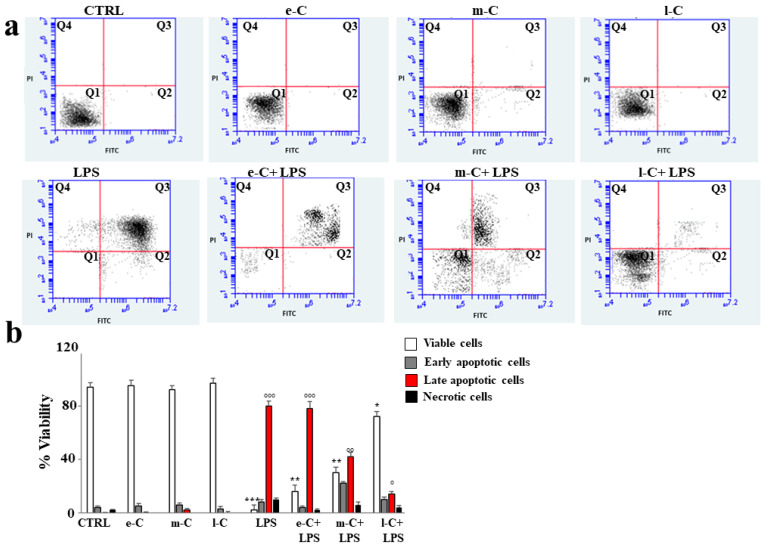
Effects of extracts of *O. ficus-indica* on cellular apoptosis. To evaluate the effect of extracts of *O. ficus-indica* on the apoptotic process, citofluorimetric experiments with annexin V/PI were conducted. In (**a**), a representative experiment of three independent experiments is reported. Each box is divided into four quadrants (Q1, Q2, Q3, and Q4). Q1 refers to annexin V-negative/PI-negative cells (viable cells). Q2 refers to annexin V-positive/PI-negative cells (early apoptosis). Q3 refers to annexin V-positive/PI-positive cells (late apoptosis); Q4 refers to annexin V-negative/PI-positive cells (necrosis). In (**b**), the respective quantification is shown. Three independent experiments were performed, and the values are expressed as the mean ± sd. *** denotes *p* < 0.001 vs. Q1 of the control; ** denotes *p* < 0.01 vs. Q1 of the control; * denotes *p* < 0.05 vs. Q1 of the control; °°° denotes *p* < 0.001 vs. Q3 of the control; °° denotes *p* < 0.01 vs. Q3 of the control; ° denotes *p* < 0.05 vs. Q3 of the control. The Analysis of Variance (ANOVA) was followed by the Tukey–Kramer comparison test.

To evaluate the effect of the extracts in a model of induced inflammation, the cells were treated with lipopolysaccharide (LPS, 1 µg/mL for 24 h), one of the components of the outer layer of the cell wall of Gram-negative bacteria, capable of causing strong immune responses. As can be seen in Figure 7a, the annexin/PI test was used to assess viability or type of cell death resulting from appropriate treatments. Prolonged cellular treatment with LPS induces irreparable damage that can be assessed as apoptotic death. Untreated cells or those exposed only to the extracts of cladodes (e-C, m-C, and l-C) showed no death and the cells appeared fully viable. Apoptotic cell death induced by LPS is evidenced by the shift of cells from Q1 to Q3. Surprisingly, pre-treatment with l-C, before exposure to LPS, resulted in substantial cellular protection visible through reduced apoptosis and the enrichment of viable cells. Pre-treatment with e-C did not reduce cell death at all, while m-C generated an intermediate effect between e-C and l-C, reducing apoptosis and increasing cell viability; however, both variations were only slight. In panel b, the respective quantification is shown.

### 2.7. O. ficus-indica Extracts and Caspases

**Figure 8:**
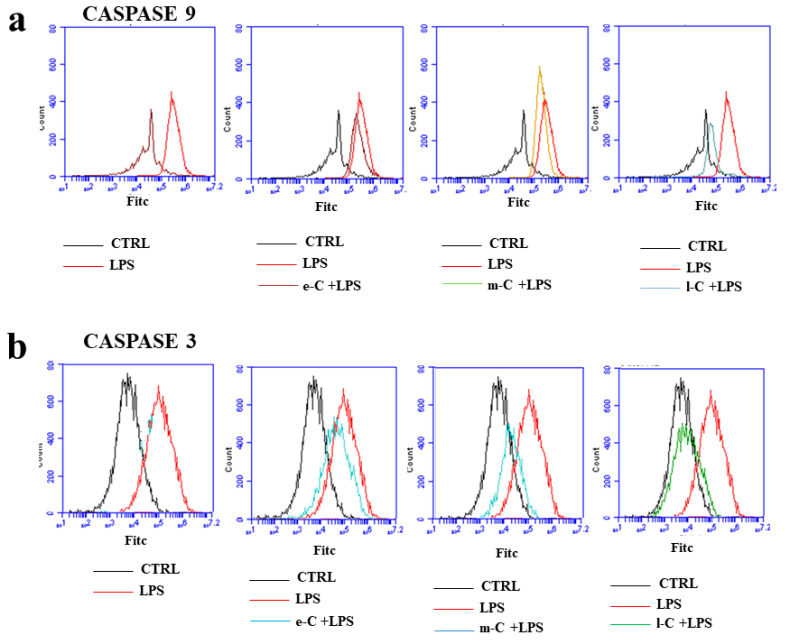
Effects of extracts of *O. ficus-indica* on caspases. Monoclonal antibodies linked to fluorochromes were used to measure the expression of caspase 9 (**a**) and caspase 3 (**b**). A representative experiment of three independent experiments is shown.

The expression of two of the main apoptotic markers (cleaved fraction of caspase 9 and 3) was evaluated by cytofluorescence, as can be seen in Figure 8a,b, respectively. In the first box of panel a, the difference in expression of caspase 9, between untreated cells and those exposed to LPS, is highlighted. Inflammatory treatment caused a shift of fluorescence to the right, which can be interpreted as an increased expression of the apoptotic marker. While e-C and m-C could not revert the increased expression of caspase 9, the l-C extract is the only one that significantly reduced LPS-induced damage. In panel b, the expression of cleaved caspase 3 is shown. In this case, pre-treatment with e-C and m-C already reduced the expression of caspase, but l-C shows expression levels similar to those of the untreated cells. In summary, it is possible to say that in the case of induced apoptosis (Figure 7 and Figure 8), l-C extract is the one that works most significantly, reducing the induced damage.

### 2.8. Determination of Total Phenolic and Flavonoid Content

**Figure 9:**
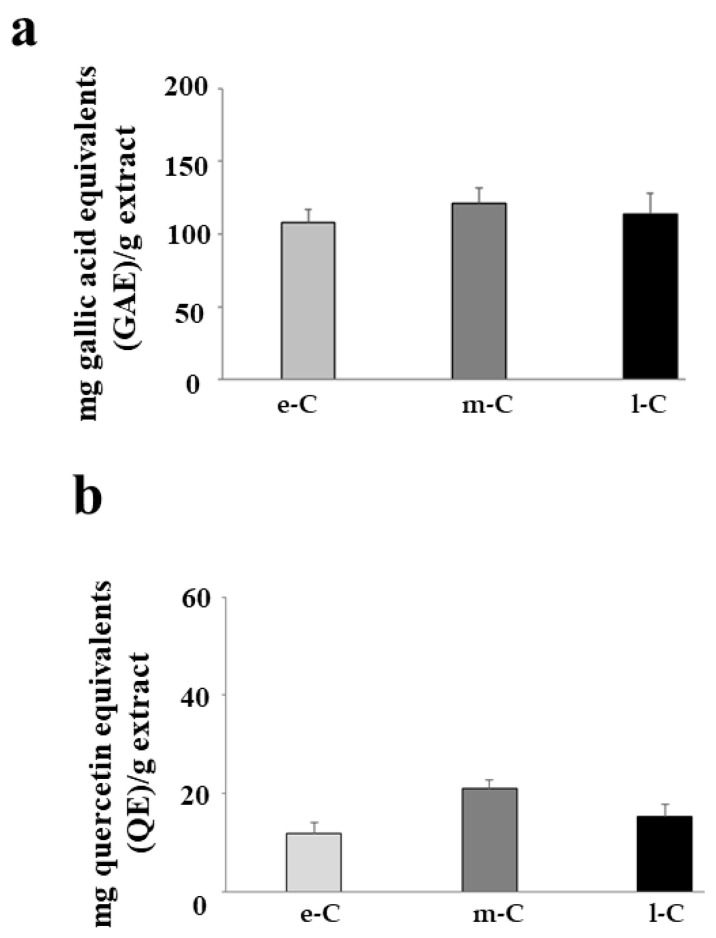
Total polyphenol and flavonoid content. The polyphenol content of the extracts e-C, m-C, and l-C is shown in (**a**). Values are expressed as mg gallic acid equivalents/g extract. In (**b**), the levels of flavonoids are represented, and the values are expressed as mg quercetin equivalents/g extract. Three independent experiments were performed, and the values are expressed as the mean ± sd.

Figure 9a,b show the total content of polyphenols and flavonoids, respectively, present in the tested extracts. As can be seen in the figure, the extracts e-C, m-C, and l-C have the same amount of both polyphenols and flavonoids. The absence of a significant difference in the content of polyphenols and flavonoids (in the extracts under consideration) indicates that the reasons for the results obtained should not be found in quantitative differences between these compounds.

## 3. Discussion

The first important result of this work was making the extracts (e-C, m-C, and l-C) less toxic: the literature indicated as preferential protocol their dissolution in a hydroalcoholic solution consisting of water and methanol (ratio 20:80) [37,38]. However, the presence of methanol increased the level of toxicity and the risk of not being able to administer e-C, m-C, and l-C at high concentrations. Methanol is the simplest of the alcohols and its chemical formula is CH_3_OH; it is very volatile, extremely flammable, and toxic, inducing depression of the central nervous system and damage to the optic nerve and retina [39]. Ethanol (CH_3_CH_2_OH) is very similar to methanol but contains one carbon and two other hydrogen atoms in its molecule. Despite the minimal structural difference, ethanol is less toxic and safer than methanol [40]. The lower toxicity of ethanol led us to replace methanol with this alcohol. However, the simple substitution was not sufficient to guarantee the same rate of solubility and it was necessary to help the dissolution process by increasing the temperature up to 30 °C.

The results obtained in this manuscript indicated that specific stages of maturity of the cladodes of *O. ficus-indica* influence their aptitude for specific properties. For example, m-C has high antioxidant properties and this potential is significantly reduced in both e-C and l-C. Similarly, l-C has a more consistent anti-apoptotic activity than e-C and m-C, showing that the different stages of maturity determine greater or lesser effects of extracts.

The antioxidant activity can presumably be carried out by polyphenols, as widely demonstrated, and they are particularly abundant in cladodes [41,42,43]. It is important to remember that plants are known to be sources of antioxidant compounds and that their amounts depend on a variety of conditions that can increase or reduce their content, such as climate, soil, and stress factors, among others [44,45]. The observed apoptotic activity is a consequence of an inflammatory stimulus-induced process: the cells were treated with LPS (1 µg/mL for 24 h) and the prolonged pro-inflammatory process caused irreversible damage resulting in apoptotic death. Polyphenols also seem to be widely involved in inflammatory protection [46,47] and, therefore, they could be responsible for the activity carried out by l-C. Given these considerations, it would be expected that the polyphenolic content would be different in all the fractions considered and probably higher in m-C and l-C. On the contrary, surprisingly the content of polyphenols and flavonoids was unchanged in e-C, m-C, and l-C. The only explanation capable of justifying this phenomenon is that a qualitative variation of polyphenols occurs instead of a quantitative one. To this end, it is likely that m-C is made up of polyphenols that perform more antioxidant functions, and better, while l-C would have a polyphenolic content more capable of performing anti-inflammatory and anti-apoptotic activity; this substantial qualitative difference would not involve the amount of polyphenolic content. It is important not to forget that polyphenols are secondary metabolites of plants that play a key role in both their defense and signaling [48] by organizing defense mechanisms against herbivores, pathogenic bacteria, fungi, viruses, and parasites, developing tolerance to various forms of stress and fostering relations with pollinating insects [49]. Cladodes are therefore likely to produce specific polyphenols depending on the stage of maturity. Another hypothesis capable of explaining the qualitative polyphenolic variations of plants is that some physical characteristics (the age of the plant or seasonality) can modulate gene expression, increasing the formation of specific secondary metabolites [50,51].

## 4. Conclusions

The results obtained appear interesting and promising, although further research needs to be conducted on this subject. In addition to the in vitro study, it would be desirable to conduct in vivo experiments, so that all physiological reactions and processes can be included. The polyphenols allegedly explain the antioxidant effects of the extracts and to this end investigations on the bioavailability of these compounds, following ingestion, digestion, and expulsion, could be conducted. The anti-apoptotic effect resulting from an induced inflammatory stimulus could be the starting point for using these extracts in inflammatory degeneration. If the results obtained are confirmed and our assumptions are true, the use of *O. ficus-indica* could greatly increase and this plant could guarantee specific remedies for human health. Finally, it would be desirable to quickly identify which polyphenols are directly responsible for the responses observed.

## 5. Materials and Methods

### 5.1. Plant Materials

The cladodes of *Opuntia ficus-indica* were collected at Roccelletta di Borgia, Calabria, Italy (February 2023, temperature: 10 °C): latitude 31, 57′22″ N, longitude 12, 23, 21′38″ E, from the same plant, but with different degrees of maturity. The taxonomic identification of the plant was confirmed by S. Ragusa, full professor of Pharmaceutical Botany, Department of Health Sciences, University “Magna Graecia” of Catanzaro, Italy. It was useful to distinguish between early, medium, and late cladodes. To respect this condition, some parameters were chosen such as the number of days of presence on the plant, the size, the different weights, and the length. These parameters are reported and listed in Table 1.

### 5.2. Sample Preparation

The chosen cladodes were collected and washed with distilled water; the plugs were removed and cut into pieces of around 1 cm. Subsequently, the cladodes were dried in a laboratory oven (ENCO, Venice, Italy), at a temperature of 40 °C for 4 days; the dried pieces were ground (2000 W, velocity 25,000 r/min, running time 5 min with an interval time of 10 min) and then sieved through 200 µm mesh sieves to obtain a green powder with an intermediate granulometry. The powder obtained was stored in an airtight glass bottle at 4 °C, away from light and moisture, until further use [23]. At the time of use, the powder was dissolved in a solution of water and ethanol (20:80), at 30 °C, and the mixture obtained was stirred for 2 h in the dark. Using this approach, three extracts were obtained and tested: e-C, m-C, and l-C.

### 5.3. Determination of Total Phenolic and Flavonoid Content

The total content of polyphenols in the extracts was calculated using the Folin–Ciocalteu colorimetric assay. First, several solutions of gallic acid with different concentrations (0, 25, 50, 100, 200, and 300 µg/mL) were used. Subsequently, 1 g of powdered cladodes of *O. ficus-indica* was mixed with 20 mL ethanol–water 80:20 (*w*/*w*) and this mixture was shaken for 24 h. An amount of 400 μL of the mixture was put in one cuvette and 0.8 mL of Folin–Ciocalteu reagent diluted 10 times was added. After 3 min of stirring, 0.8 mL of sodium carbonate 7% (*w*/*v*) was added and the resulting mixture was left to stand for a further 2 h and stirred constantly until color developed. The relative absorbance was measured at 760 nm with a Prism V-1200 spectrophotometer and the phenolic content was determined by the linear equation of the standard curve. The total phenolic content of the extract was expressed in mg Gallic acid equivalent (GAE)/g dry weight. For determination of total flavonoid content, the aluminium-chloride colorimetric test was used. An amount of 1 mL of extract was mixed with 1 mL of 2% aluminum chloride in methanol. After 30 min, the absorbance at 430 nm was measured and quercetin equivalents per gram of extract (mg QE/g extract) were used to represent the estimated flavonoid content.

### 5.4. Antioxidant Activity

#### 5.4.1. The ORAC Assay

The antioxidant activity of e-C, m-C, and l-C was determined by ORAC assay. The loss of fluorescence of fluorescein (used as a probe) is measured over time and this fluorescence is due to the formation of peroxylic radicals, following the spontaneous degradation of 2,2′-azobis-2-methyl-propanimidamide, dihydrchloride (AAPH). The peroxylic radical oxidizes the fluorescein, causing the gradual loss of the fluorescence signal. Antioxidants suppress this reaction and inhibit signal loss: 6-hydroxy-2,5,7,8-tetramTethylchroman-2-carboxylic acid (Trolox) is a water-soluble analog of vitamin E that inhibits fluorescence decay in a dose-dependent manner. Finally, e-C, m-C, and l-C were used at a concentration of 0.02 mg/mL. The evaluation of fluorescent decay for fluorescein was conducted using 485 and 520 nm as excitation and emission wavelengths, respectively. The temperature was maintained at 37 °C. Measurements were made in triplicate, and data obtained from fluorescence vs. time curves are reported as the average antioxidant efficacy of the antioxidant compound. A regression equation was constructed by comparing the net area below the fluorescein decay curve and the Trolox concentration. The area under the curve was calculated with the following equation:

i = 90

AUC = 1 + Σ f1/f1

i = 1

#### 5.4.2. DPPH Test

The antioxidant activity of the extracts obtained from the cladodes of *O. ficus-indica* was measured using the stable radical 2,20-difenyl-1 picrylhydrazyl (DPPH). Experimentally, 850 µL of DPPH solution was added to 50 µL of various extract concentrations (0.02–0.25 mg/mL), keeping the mixture in the dark for 20 min. Subsequently, the absorbance was read by a UV–Vis spectrophotometer (Multiskan GO, Thermo Scientific, Denver, CO, USA) at 517 nm, room temperature, and its reduction in absorbance was visible as a change in color from purple to yellow. The results obtained were expressed in % inhibition and IC_50_. The latter represents the concentration of matter in powder required to remove 50% of DPPH radicals.

### 5.5. Cell Cultures

The Human Endothelial Cell line (Huvec) had been isolated from the vein of an umbilical cord and was acquired from the American Type Culture Collection (ATCC, 20,099 Sesto San Giovanni, Milan, Italy). Huvec cells were grown in culture in modified Dulbecco’s Eagle medium (DMEM), (enriched with 100 U/mL penicillin, 100 µg/mL streptomycin, and 10% fetal bovine serum) and kept in a humidified 5% CO_2_ atmosphere at 37% C. The medium was changed every 2–3 days and when the cell lines reached 50% of the confluence, they were treated with e-C, m-C, and l-C (0.02 mg/mL) for 24 h or pre-treated with these extracts (0.02 mg/mL) for 24 h and then exposed to H_2_O_2_ 50 µM for 9 h or to LPS 1 µg/mL for 24 h.

### 5.6. Proliferation Assay

Cell proliferation was measured through an MTT test; this dye, when contained in living cells with functioning mitochondria, can be reduced, resulting in colorimetric variation. MTT reduction measurement provides information on cell viability and metabolic activity. A total of 8 × 10^3^ cells were plated in 96-well plates and, after 24 h, the culture medium was replaced with a fresh medium containing e-C, m-C, or l-C for 24 h. At the end of the treatment time, the medium was replaced with a phenol-free medium containing a solution of MTT (0.5 mg/mL) and after 4h incubation, 100 µL of 10% SDS was added to each well to solubilize the formazan crystals. Finally, the optical density was measured at wavelengths of 540 and 690 nm using a spectrophotometer reader (X MARK Microplate Bio-Rad Spectrophotometer, Bio-Rad, Hercules, CA, USA).

### 5.7. ROS Measurement in the Huvec Cell Line

The probe 2′,7′-dichlorodihydrofluorescein diacetate (H_2_DCF-DA) can be used to measure reactive oxygen species accumulated in cells, taking advantage of its peculiarities: (1) it easily enters cells: (2) H_2_DCF-DA is cleaved, by intracellular esterase, to form H_2_DCF; (3) H_2_DCF is no longer able to leave the cells; (4) if H2DCF is oxidized, it binds to the ROS, forming the compound highly fluorescent DCF. The quantification of the DCF probe provides the content of ROS in the cell. Experimentally, 6 × 10^4^ cells were plated in 96-well microplates, and the following day, Huvec cells were pre-treated with e-C, m-C, or l-C for 24 h at the concentration of 0.02 mg/mL for 24 h. At the end of the treatment, the growth medium was replaced by a phenol-free fresh medium containing H_2_DCF-DA (25 µM) and, after 30 min of exposure to 37 °C, the cells were washed with PBS, centrifuged, resuspended in PBS, and then exposed or not at H_2_O_2_ (100 µM, 20 min). Finally, the fluorescence was evaluated by cytometric analysis (FACS Accury, Becton Dickinson, Franklin Lakes, NJ, USA).

### 5.8. Annexin V/PI Staining

After treating the cells as previously indicated, they are trypsinized, washed with PBS, and then the Huvec cells were resuspended in 1x binding buffer (Metabolic Activity/AnnexinV/Dead Cell Apoptosis Kit). One hundred microliters of the suspension was transferred to a 5 mL culture tube and 5 µL of FITC annexin V (BD Biosciences, San Jose, CA, USA) was added. The samples were vortexed and incubated for 15 min in the dark, at 25 °C. Finally, 400 µL of 1 binding buffer and 5 µL of propyl iodide (PI) were added to each tube and samples were analyzed with flow cytometry (Accury FACS, Becton Dickinson laser cytometer).

### 5.9. Measurement of Protein Expression through Immuno-Cytofluorometry

To measure the expression of caspases 9 and 3, monoclonal antibodies, linked to fluorochromes, were used and the samples were analyzed through cytofluorometry. The cells were washed with PBS, trypsinized, and then collected in a growth medium in cytofluorometer tubes. Subsequently, they were treated with BSA in 10% animal serum and kept for 60 min at room temperature to block non-specific sites. At the end of the indicated time, the cells were incubated for 2 h at 37 °C with the desired antibodies. For caspase 9, a rabbit monoclonal antibody (2D5) was purchased from Invitrogen, at 1:200 dilution. For caspase 3, a rabbit monoclonal antibody (E87) was purchased from Abcam (197202) at 1:200 dilution. After appropriate washing with PBS to remove excess primary antibodies, secondary antibodies, conjugated with Fitc, diluted in 5% animal serum for 1 h at room temperature, and 1:500 dilution, were added. After further washing in PBS, the cytofluorometer reading was carried out.

## Figures and Tables

**Table 1:** Characteristics of e-C, m-C, and l-C.

	Early Cladodes	Medium Cladodes	Late Cladodes
Days	40	100	150
Grams	150–220	280	415
cm	3–10 cm	11–25 cm	26–50 cm

## Data Availability

All data available within the manuscript.
